# Characterization of Systemic Oxidative Stress in Asthmatic Adults Compared to Healthy Controls and Its Association with the Oxidative Potential of Particulate Matter Collected Using Personal Samplers

**DOI:** 10.3390/antiox14040385

**Published:** 2025-03-25

**Authors:** Miguel Santibáñez, Adriana Núñez-Robainas, Esther Barreiro, Andrea Expósito, Juan Agüero, Juan Luis García-Rivero, Beatriz Abascal, Carlos Antonio Amado, Juan José Ruiz-Cubillán, Carmen Fernández-Sobaler, María Teresa García-Unzueta, José Manuel Cifrián, Ignacio Fernandez-Olmo

**Affiliations:** 1 Global Health Research Group, Departamento Enfermería, Faculty of Nursing, Universidad de Cantabria-IDIVAL, Avda. Valdecilla, s/n, 39008 Santander, Spain; 2 Centro de Investigación en Red de Enfermedades Respiratorias (CIBERES), Instituto de Salud Carlos III (ISCIII), 08003 Barcelona, Spain; adriananunez96@gmail.com (A.N.-R.); ebarreiro@imim.es (E.B.); 3 Pulmonology Department-Muscle Wasting and Cachexia in Chronic Respiratory Diseases and Lung Cancer, IMIM-Hospital del Mar, Department of Medicine and Life Sciences (MELIS), Universitat Pompeu Fabra (UPF), Barcelona Biomedical Research Park (PRBB), 08003 Barcelona, Spain; 4 Departamento de Ingenierías Química y Biomolecular, Universidad de Cantabria, Avda. Los Castros, s/n, 39005 Santander, Spain; andreaexpositomonar@gmail.com (A.E.); ignacio.fernandez@unican.es (I.F.-O.); 5 Division of Pneumology, Hospital Universitario Marqués de Valdecilla, IDIVAL, 39008 Santander, Spain; jagcal@gmail.com (J.A.); jgarcia@separ.es (J.L.G.-R.); beatriz.abascal@scsalud.es (B.A.); amadodiago.carlos@gmail.com (C.A.A.); juanjose.ruizc@scsalud.es (J.J.R.-C.); josemanuel.cifrian@scsalud.es (J.M.C.); 6 Division of Biochemistry, Hospital Universitario Marqués de Valdecilla, IDIVAL, 39008 Santander, Spain; sobaler@mirapeix.org (C.F.-S.); mteresa.garciau@scsalud.es (M.T.G.-U.)

**Keywords:** particulate matter (PM), oxidative potential (OP), asthma, systemic oxidative stress, OxLDL

## Abstract

Inflammatory cell activation in asthma may lead to reactive oxygen species (ROS) overproduction with an imbalance between oxidant levels and antioxidant capacity, called oxidative stress (OS). Since particulate matter (PM) airborne exposure may also contribute to ROS generation, it is unclear whether PM contributes more to OS than inflammatory cell activation. In our ASTHMA-FENOP study, which included 44 asthma patients and 37 matched controls, we aimed to characterize OS using five serum markers: total ROS content, protein carbonyl content, oxidized low-density lipoprotein (OxLDL), 8-hydroxydeoxyguanosine, and glutathione. Volunteers wore personal samplers for 24 h, collecting fine and coarse PM fractions separately, and the oxidative potential (OP) was determined using two methods. We observed differences between asthmatic and non-asthmatic volunteers in some OS markers, such as OxLDL, with an adjusted mean difference of 50,059.8 ng/mL (*p* < 0.001). However, we did not find an association between higher PM-OP and increased systemic OS. This suggests that at our PM-OP exposure levels, OS generated by the inflammatory cells themselves is more relevant than that generated by airborne PM. This supports the idea that asthma is a heterogeneous disease at the molecular level, mediated by inflammatory cell activation, and that OS may have potential clinical implications.

## 1. Introduction

Among the air pollutants with a greater impact on health, airborne particles or particulate matter (PM) are the most relevant, especially the finest ones (fine fraction or PM2.5) since they have the ability to access the alveolus and the bloodstream [1,2,3,4]. This negative impact on health is not only due to the mass concentration of PM but also fundamentally due to its chemical composition and more specifically and accurately with its capacity to generate reactive oxygen species (ROS) in cells, altering the oxidant/antioxidant balance and causing oxidative stress [5]. These ROS are known for their ability to oxidize lipids and proteins, as well as damage DNA and RNA, resulting in increased airway and systemic inflammatory response, which can lead to various diseases, mainly respiratory [6,7] with a relevant role in asthma [8,9,10,11,12,13,14,15,16,17,18]. Therefore, a global parameter that indicates the oxidation capacity of PM components can be used as an alternative representative metric of air quality with greater implications for human health. One parameter of these characteristics is the oxidative potential of PM (PM-OP), which measures the ability of PM components to either oxidize or catalyze the oxidation of antioxidant target molecules that are present in biological fluids, such as ascorbic acid (AA), or that act as substitutes for these molecules, such as dithiothreitol (DTT), leading to the simultaneous production of ROS [8,19].

In epidemiological studies, the characterization of PM exposure is mainly based on stationary samplers rather than individual measurements. Using personal PM samplers instead of stationary ones allows for the collection of particles to which a volunteer has been exposed in the last 24 h. These personal PM samplers have been used in some studies to determine the mass and chemical composition of PM [20,21], and more recently, its OP [8,22,23,24,25].

Asthma is the most common chronic respiratory disease in the world, affecting 4.7% and as mentioned above, air pollution plays a role in both its onset and worsening [26,27,28,29,30], with growing evidence that an important pathogenic feature in this disease is the imbalance between ROS and antioxidant capacity that leads to oxidative stress. Chronic inflammation in asthma is mediated by an activation of macrophages, neutrophils, and eosinophils, with an invasion of the bronchial mucosa of these inflammatory cells. In addition to the ROS incorporated by the airborne PM, activated macrophages, neutrophils, and eosinophils are able to release ROS [11,12,13]. Therefore, it is not clear whether, in terms of ROS, the contribution of PM inhalation is greater than that of the inflammatory cells themselves. What does seem clearer in translational terms is that certain systemic markers of oxidative stress could potentially be biomarkers of asthma activity and control, as oxidative stress would ultimately result in increasing bronchial hyperactivity in a vicious circle [6,7].

In our ASTHMA-FENOP exploratory study, we aimed to characterize systemic oxidative stress in a sample of adult asthmatic patients and healthy controls (without asthma) through five oxidative stress markers and to study the potential association between higher levels of individual exposure to PM-OP using personal samplers and higher oxidative stress.

## 2. Methods

### 2.1. Study Design

The study design has been described elsewhere [31]. Briefly, we conducted a cross-sectional study on 44 adult asthma patients and 37 controls matched with asthma patients by gender, age (±5 years old), and smoking status (never, former). Most of the volunteers lived in the Santander urban area, and a second subgroup lived in the Maliaño area (Camargo) (near some metallurgical plants), constituting an urban–industrial mixed area. In both areas, a former stationary PM sampling campaign was conducted [32]. The locations of both stationary sampling sites and the volunteers’ residences are depicted in Figure S1. Inclusion and exclusion criteria are shown in Table S1. Controls underwent a review of the diagnoses reflected in their clinical history and an examination by a pneumologist. None of them had chronic or recurrent respiratory symptoms or features typical of asthma.

### 2.2. Recruitment Scheme and PM Personal Sampling

The recruitment scheme involved three consecutive days, with 1–4 patients per week from November 2022 to May 2023. Recruitment was conducted in collaboration with the Pneumology Service of Hospital Universitario Marqués de Valdecilla (HUMV) and Hospital de Liencres (HL). After signing the informed consent form, each volunteer received a PM personal sampler on arrival on the first day (visit 1) to be wore for at least 24 h. We used two-stage personal modular impactors (SKC PMI coarse) capable of sampling PM2.5 and PM10-2.5 filters separately, connected to a personal pump (SKC Aircheck XR5000, SKC Inc., Valley View Road Eighty Four, PA, USA) that operated at a flow rate of 3 L per minute. Here, 37 and 25 mm diameter polytetrafluoroethylene (PTFE) membrane filters were used for PM2.5 and PM10-2.5 fractions, respectively. On day 3 (lag1, 25–48 h after returning the personal sampler), fractional exhaled nitric oxide (FeNO) determinations were conducted, and a blood sample was obtained in order to determine the oxidative stress biomarkers. The protocol for each volunteer is summarized in Table S2.

### 2.3. Oxidative Potential Analysis

The PM2.5 and PM10-2.5 filters (fine and coarse fractions) were extracted with 5 mL of a phosphate-buffered solution (0.0075 M Na_2_HPO_4_, 0.0025 M NaH_2_PO_4_) for 24 h at 37 °C and filtered using a syringe cartridge. The samples were stored until OP analysis at 4 °C.

Two OP assays were carried out based on the methodology developed by Expósito et al. [33]: DTT and AA assays. A microplate reader spectrophotometer (Multiskan Skyhigh microplate spectrophotometer, Thermo Fisher Scientific Inc., Singapore) was used for the OP measurements. Further details of the OP assays procedures have been published [33]. Samples were analyzed in triplicate. The detection limit calculation was described previously in detail [31]. The calculations are shown in Table S3.

### 2.4. Oxidative Stress Measurement

Fasting blood samples were collected from all participants at visit 3 (from 8:00 to 9:00 a.m.). A standard blood count, conventional lipid profile including total low-density lipoprotein (LDL), and glucose determinations were protocolized. In these blood extractions, serum was then separated and stored at −80 °C until assayed. The quantification of oxidative stress was performed for all serum samples at the same time utilizing OxiSelect^TM^ ELISA kits (Cell Biolabs, Inc., San Diego, CA, USA) for the first four oxidative stress markers: in vitro ROS/reactive nitrogen species (RNS), protein carbonyl content (PCC), oxidized LDL (OxLDL) in the form of 4-hydroxynonenal-modified LDL (HNE-OxLDL), and 8-hydroxydeoxyguanosine (8-OHdG). And the human reduced glutathione (GSH) ELISA Kit (MyBioSource, San Diego, CA, USA) was used for the last marker, following, in all cases, the manufacturer’s instructions. A detailed description of the ELISA determinations is shown in the Supplemental Information (Text S1).

Table S4 describes our 5 different oxidative stress measurements, indicating the specific ELISA kit used, intra-assay coefficient ranges, and lowest and highest quantifications, as well as the studies that have used them in blood samples [1,34,35,36,37,38,39,40,41,42,43,44,45].

### 2.5. Statistical Analysis

Continuous variables were described using the mean and standard deviation (SD) and/or median and interquartile ranges (IQR). Statistical differences between groups were compared using Student’s *t*-test (for equal or different variances, depending on the results of the Levene test) for mean comparisons. Medians were compared using the Mann–Whitney U test. Categorical and discrete variables were expressed as percentages, and comparisons were performed using the Chi-square test, with Yates’ correction or Fisher’s exact test, as appropriate.

Oxidative stress markers and PM-OP exposure metrics levels were dichotomized based on their medians. Crude and adjusted odds ratios (aORs) with 95% confidence intervals (CIs) were estimated using unconditional logistic regression models. In these models, the binary outcomes (0 = lower values; 1 = higher values) of each oxidative stress marker were treated as dependent variables, while binary PM-OP exposures were treated as independent binary variables.

In a parallel approach, adjusted mean differences (aMDs) with their 95%CI were calculated using a linear regression model in which the quantitative results for each oxidative stress marker were treated as the dependent variable, and each PM-OP exposure as a binary variable.

Asthma and control statuses, age (as a continuous variable), sex, education level (ordinally categorized), and body mass index (BMI) were pre-established as confounders to obtain adjusted ORs and MDs. A stratified analysis based on asthma and non-asthma statuses was pre-established, along with an additional multivariate model for asthma patients. This model included results from the Asthma Control Test (ACT), Test of Adherence to Inhalers (TAI), and asthma severity (GINA 2023 guideline steps) as confounders.

The level of statistical significance was set at 0.05, and all tests were two-tailed. Statistical analyses were performed using the SPSS statistical software package version 22.0 (SPSS, Inc., Chicago, IL, USA).

## 3. Results

### 3.1. Description of the Sample

The characteristics of asthma patients and controls without asthma are summarized in Table 1.

The average age of the participants was 52.26 years (SD = 16.99), with similar mean ages for both asthma patients (mean = 52.03) and controls (mean = 52.45) due to matching. Among the participants, 56.8% were women and 43.2% were men. Most volunteers were non-smokers (77.8%). The level of university education differed between the asthmatic and control groups, with a higher prevalence of university education among controls (*p* < 0.001). Regarding BMI, 36.4% and 56.8% of asthmatic and non-asthmatic volunteers were classified as having a healthy weight according to WHO standards (cutoff points, 18.5–24.9). The prevalence of overweight (BMI 25–29.9) and obesity (BMI ≥ 30) was slightly higher among asthmatic volunteers (*p* = 0.096). In contrast, the prevalence of hypercholesterolemia and total LDL values above 100 was slightly higher in non-asthmatic volunteers (*p* = 0.430 and 0.169, respectively).

Additional clinical characteristics of the asthmatic volunteers are summarized in Table S5. Most of the asthmatic participants (n = 25, 56.8%) were in GINA stage 4, receiving medium-dose maintenance prescriptions of inhaled corticosteroids (ICS)-long-acting β adrenoceptor agonists (LABAs). Adherence to their inhaled maintenance therapy, as measured by TAI, was good in the majority of patients (n = 33/44, 75.0%). Their mean score on the ACT was 22.16 points (SD = 3.8). Based on the ACT scores, 81.8% had their asthma controlled (≥20 points).

### 3.2. Description of Oxidative Stress Markers and PM-OP Level Results

The total ROS/RNS content measured as μM hydrogen peroxide (H_2_O_2_) equivalents was higher among controls (contrary to our hypothesis) with statistically significant *p* values. In contrast, levels for HNE-OxLDL as an indicator of lipid peroxidation were higher among asthmatic volunteers. PCC as an indicator of protein damage was similar among asthmatic and non-asthmatic volunteers.

The distribution of 8-OHdG as an indicator of DNA/RNA damage and repair and GSH as indicator of the antioxidant capacity presented positive asymmetry with the mean values greater than the medians. Higher levels of 8-OHdG (non-statistically significant) and lower levels of GSH (statistically significant in the comparison of medians with the Mann–Whitney U test) were found in asthmatic volunteers. See Table 2.

With respect to PM-OP metrics, PM-OP levels were higher among asthma patients compared to controls, reaching statistical significance in some cases, and indicating a higher PM-OP personal exposure among asthmatics. See Table 2. The spatial distribution of PM-OP values is illustrated in Figure S1. The PM-OP levels from the personal sampling campaign did not follow a spatial pattern based on the locations of volunteers’ residences in terms of proximity to the urban and urban–industrial mixed ambient sites referenced in Section 2.1.

Table 3 shows the crude and adjusted mean differences between asthmatic and non-asthmatic volunteers for each of the oxidative stress markers in an attempt to minimize confounding bias. Mean levels of total ROS/RNS content remained statistically significant. For 8-OHdG, MDs increased with respect to the crude MD after adjusting for the predefined confounding variables, reaching statistical significance in model 3: aMD = 5.65, *p* = 0.044. Non-statistically significant aMDs were obtained for GSH. In contrast, aMDs for HNE-OxLDL remained statistically significant, even after adjusting for PM-OP metrics, FeNO and total LDL levels (aMD = 50,059.8 ng/mL *p* < 0.001). See Table 3.

### 3.3. Adjusted Associations Between PM-OP and Oxidative Stress Markers

Non-statistically significant mixed associations were observed, with aORs both below and above 1, and aMDs both positive and negative, without clear evidence of associations. See Table 4 and Table 5. These findings remained similarly mixed and non-statistically significant when analyses were restricted to either asthmatic or control volunteers separately.

## 4. Discussion

We have not found an association between higher PM-OP personal exposure and higher oxidative stress at the systemic level, with non-higher levels of total ROS/RNS content measured as μM H_2_O_2_ equivalents; PCC as a protein damage marker; OxLDL as marker of membrane lipid peroxidation; 8-OHdG as DNA/RNA damage; and non-lower levels of glutathione as an indicator of the antioxidant capacity.

A key strength of our study is the use of personal PM samplers to characterize exposure, in contrast to most studies that used stationary outdoor PM samplers, in addition to the use of size-segregated PM samples (fine and coarse fractions separately). As noted in the introduction, very few studies have used personal samplers to characterize PM-OP [8,22,23,24,25], and to our knowledge, only two studies (both in children) have used PM personal samplers in asthma patients but without measuring PM-OP [46,47]. Therefore, our study is the first one to explore the association between PM-OP obtained using personal samplers and oxidative stress levels in asthmatic patients.

Our recruitment period comprised November 2022 to May 2023. This is a period without limitations in terms of COVID-19 restrictions or use of masks. From November to May, our region experienced a temperate oceanic climate with mild, rainy winters and cool, relatively rainy springs. These fairly stable climatic conditions during the study period make the existence of differential weather conditions between asthmatic patients or controls improbable. On the other hand, since the airborne sampling was personal, both outdoor and indoor exposures were collected through personal samplers. Our previous PM-OP results using stationary ambient samplers showed higher levels of PM-OP and metals in an urban–industrial site compared to an urban site [32]. However, as shown in Figure S1, no spatial pattern was found in our personal samplers results, with no differences in the geographic distribution of places of residence between asthmatic and non-asthmatic volunteers. It supports that work and leisure activities (hobbies) outside the place of residence have a substantial contribution to an individual’s personal exposure. In any case, our population (both asthmatic patients and controls) is comparatively less exposed in terms of PM-OP levels than those reported in other studies [48]. One explanation for our absence of associations between PM-OP and systemic oxidative stress may be that there is not a high enough range of variability in personal exposure. It is plausible to think that with a range that includes larger personal exposures, we would perhaps have found positive associations. Another limitation is our relatively small sample size, which implies a greater role of chance in our results. Therefore, future studies with larger sample sizes and, if possible, in populations with a wider range of PM OP exposure are needed to corroborate our results.

Asthma is a long-term respiratory condition characterized by chronic inflammation, with inflammatory cell activation (macrophages, neutrophils, and eosinophils) and the expression of several mediators. This leads to increased airway sensitivity, excessive mucus production, and the shedding of epithelial cells. Regarding systemic oxidative stress, on the one hand, inflammatory cells invade the bronchial mucosa releasing by themselves ROS, e.g., hydroxyl radicals, superoxides, and H_2_O_2_ [11,12,13]. This involves an imbalance of the levels of oxidants/antioxidants in cells, causing oxidative stress [14,37,49,50]. On the other hand, hypothetically, environmental exposure to PM involves the entry of PM-bound ROS or PM components with the ability to generate ROS, altering (in a combined action with the ROS from the inflammatory cells) the mentioned balance of oxidants/antioxidants, causing more oxidative stress and leading to more chronic inflammation in a vicious cycle [8,9,10,14,15,16,17,18]. In spite of the limitations described above, our results suggest that at our PM exposure levels, oxidative stress generated by the inflammatory cells themselves is more important than that generated by the inhaled PM.

OxLDL is recognized as a major parameter involved in the pathogenesis of atherosclerosis [51,52]. It is also suggested that it may interact with granulocytes (neutrophils, eosinophils, basophils) in airway diseases like asthma, so LDL oxidation might be an important mediator for the initiation of bronchial inflammation when granulocytes are recruited to the lungs [51,52,53,54]. It is well known that LDL oxidation is initiated by free radicals. Trace elements like iron (Fe) and copper (Cu) may contribute to LDL oxidation by favoring the catalysis of lipid peroxidation. In this regard, it has been found that plasma OxLDL, Cu, and Fe levels were significantly higher in asthmatic patients compared to controls [41], and OxLDL has been modified by in vitro incubation with cigarette smoke or Cu ions [55]. On the other hand, human serum paraoxonase-1 (PON-1) is a critical antioxidant defense system against lipid oxidation. Decreased PON-1 activity has been associated with systemic oxidative stress in several disease states. A recently published meta-analysis has shown that serum PON-1 concentrations are significantly lower in patients with asthma, suggesting the presence of an impaired antioxidant defense in this group [56]. Our clear differences when comparing OxLDL between asthmatic and non-asthmatic volunteers support this rationale and deserve further consideration. Regarding the sensitivity of our PM-OP assays to the chemical composition of PM, although both methods (OP-DTT and OP-AA) are very sensitive to soluble Cu, and the AA method is also sensitive to Fe [1,8,33], we have not found an association between higher PM-OP and higher OxLDL levels by any of the methods.

Evidence from published studies on the rest of oxidative stress markers depends on the matrix in which oxidative markers are measured, with mixed results.

In blood samples, Karadogan et al. [37] compared plasma levels of several oxidative stress markers between allergic asthma subjects and controls. Statistically significant higher mean levels of malondialdehyde (MDA) (another marker of lipid peroxidation like OxLDL) (3.38 vs. 2.31 nmol/mL) and PCC (1.47 vs. 1.01 nmol/mg protein) as well as decreased GSH (16.41 vs. 23.74 nmol/mL) levels were observed in allergic asthmatics. In Lima (Peru), Checkley et al. [45] found lower relative concentrations of GSH in a nested case–control study of 100 children. In contrast, Wood et al. [57] found no differences between mild asthmatic volunteers (n = 15) and age- and sex-matched controls (n = 15) for plasma levels of glutathione peroxidase or superoxide dismutase, although superoxide dismutase activity was negatively associated with asthma severity and increased plasma levels of isoprostane 8-iso-PGF2alpha (a marker of in vivo oxidative stress belonging to the F2-isoprostanes subgroup). Several free radicals, antioxidant enzymes, free radical scavengers, or lipid peroxidation products were compared in blood in asthmatic and matched healthy control children in Chennai (India) by Shanmugasundaram et al. [58]. Excessive production of superoxide and hydroxyl radicals was observed in asthmatic patients, whereas superoxide dismutase and free radical scavengers were significantly lower in asthmatic children. In Poland, Bazan-Socha et al. [59] found an increased protein hydroperoxide formation using the coumarin boronic acid assay, in their 74 asthmatic subjects compared to 65 matched controls. Lastly, to our knowledge, studies including determinations of the total ROS/RNS content in blood have not been performed in asthmatic subjects. The kit used in this work (OxiSelect STA-347) measures the total ROS/RNS present in the serum samples, i.e., it may include H_2_O_2_, peroxyl radical (ROO·), nitric oxide (NO), and peroxynitrite anion (ONOO^−^), among others, but according to the manufacturer’s instructions, the standard curve is prepared with one of these species (H_2_O_2_), so results are expressed as concentration of H_2_O_2_ equivalents. Therefore, the results obtained in this work (higher levels in controls than in asthmatic patients) should be carefully interpreted, since each subject serum sample may contain different proportions of the ROS/RNS active in the assay. In fact, the use of the dichlorodihydrofluorescein (DCFH) probe, which is the basis of the STA-347 kit to measure intracellular H_2_O_2_ and other reactive oxygen species, is not straightforward because of the complex redox chemistry of DCFH and the limitations and artifacts associated with this assay [60,61]. Nevertheless, results from global biomarkers of oxidative stress are sometimes difficult to interpret. For example, the EGEA study revealed that the levels of fluorescent oxidation products (FlOPs) were lower in participants with asthma compared to controls, even after adjusting for age, sex, and smoking status [62]. FlOPs are global biomarkers of damage to oxidative stress, since they reflect a mixture of different oxidation products from lipids, proteins, and DNA [63]. What seems clear is that excessive production of NO during inflammatory responses can lead to the formation of various RNS in asthma [7]. As it is plausible that this nitrosative stress could be susceptible to improvement with treatments such as theophylline [a nonselective phosphodiesterase inhibitor that is used as a bronchodilator to treat asthma and Chronic Obstructive Pulmonary Disease (COPD)] [64], the study of NO-derived RNS in the physiopathology of asthma with proper markers and the study of potential treatments to improve nitrosative stress deserve further consideration.

In urine, an Italian multicase–control population-based study did not find differences between current asthma cases and controls in 8-OHdG, GSH, or 8-isoprostane (another oxidative stress marker, indicative of oxidative damage) [65].

In exhaled breath condensate, a systematic review identified sixteen oxidative stress articles. Concentrations of H_2_O_2_ and 8-isoprostanes were generally elevated and related to lower lung function tests in adults with asthma compared to controls. However, comparisons across these studies are challenging due to differences in methodology and the need for standardization [66].

In sputum, 8-OHdG, MDA, and 8-isoprostane have been shown to be increased from asthmatic subjects compared to non-asthmatic controls in several studies from another review [67].

## 5. Conclusions

We did not find an association between PM-OP and systemic oxidative stress. However, we observed differences between asthmatic and non-asthmatic volunteers in some oxidative stress markers, such as OxLDL. This supports the idea that asthma is a heterogeneous disease at the molecular level, for which oxidative stress may have potential clinical implications. Further studies with larger sample sizes and, if possible, a wider range of personal exposures are needed to confirm our results.

## Figures and Tables

**Table 1:** Description of sample as a function of their asthma or control statuses.

	Asthma		Non-Asthma		All		*p Value*
	N = 44		N = 37		N = 81		
Age, yrs. Mean [SD]	52.45	17.42	52.03	16.69	52.26	16.99	*0.911*
Age, yrs. Median [IQR]	50	40–69	54	39–67	52	39.5–68.5	*0.794*
Sex at birth							
Female	25	56.80%	21	56.8%	46	56.8%	*1*
Male	19	43.20%	16	43.2%	35	43.2%	
Non-smoker	34	77.3%	29	78.4%	63	77.8%	*0.905*
Former smoker	10	22.7%	8	21.6%	18	22.2%	
Study level							
Primary education	5	11.4%	1	2.7%	6	7.4%	*<0.001*
Secondary education	13	29.5%	3	8.1%	16	19.8%	
High school level	16	36.4%	2	5.4%	18	22.2%	
University studies	10	22.7%	31	83.8%	41	50.6%	
BMI (WHO classification)							
Healthy weight 18.5–24.9	16	36.4%	21	56.8%	37	45.7%	*0.096*
Overweight 25–29.9	18	40.9%	13	35.1%	31	38.3%	
Obesity ≥ 30	10	22.7%	3	8.1%	13	16.0%	
Cholesterol levels, mg/dL (Visit 3). Mean [SD]	191.2	37.76	189.54	36.17	190.44	36.82	*0.841*
Cholesterol levels, mg/dL (Visit 3). Median [IQR]	187.5	50	190	53	189	51	*0.894*
Total cholesterol > 200 mg/dL (Visit 3)							
No	31	70.5%	23	62.2%	54	66.7%	*0.43*
Yes	13	29.5%	14	37.8%	27	33.3%	
HDL levels, mg/dL (Visit 3). Mean [SD]	58.27	16.09	118.41	28.17	58.98	15.98	*0.396*
HDL levels, mg/dL (Visit 3). Median [IQR]	56	23	59	22	57	22	*0.652*
HDL levels < 60 mg/dL (Visit 3)							
No	18	40.9%	18	48.6%	36	44.4%	*0.485*
Yes	26	59.1%	19	51.4%	45	55.6%	
LDL levels, mg/dL (Visit 3). Mean [SD]	112.66	31.82	118.41	28.17	115.28	30.16	*0.396*
LDL levels, mg/dL (Visit 3). Median [IQR]	109	37	122	37	112	38	*0.172*
LDL levels > 100 mg/dL (Visit 3)							
No	17	38.6	9	0.243	26	0.321	*0.169*
Yes	27	0.614	28	0.757	55	0.679	
Diabetes or insulin resistance							
No	42	0.955	36	0.973	78	0.963	*0.662*
Yes	2	0.045	1	0.027	3	0.037	
High blood pressure							
No	35	0.795	32	0.865	67	0.827	*0.411*
Yes	9	0.205	5	0.135	14	0.173	
Atherosclerosis and cardio-vascular disease and pulmonary thromboembolism							
No	43	0.977	35	0.946	78	0.963	*0.457*
Yes	1	0.023	2	0.054	3	0.037	
Blood Eosinophils (Visit 3), cells/mm^3^, Mean [SD]	331.82	412.46	208.11	108.98	275.31	317.23	*0.062*
Blood Eosinophils (Visit 3), cells/mm^3^, Median [IQR]	200	100–400	200	100–300	200	100–300	*0.183*
Blood Eosinophils (Visit 3) ≥ 150 cells/mm^3^							
No	12	27.3%	13	35.1%	25	30.9%	*0.445*
Yes	32	72.7%	24	64.9%	56	69.1%	
Blood Neutrophils (Visit 3) ≥ 5000 cells/mm^3^							
No	37	84.1%	35	94.6%	72	88.9%	*0.134*
Yes	7	15.9%	2	5.4%	9	11.1%	
Oral corticosteroids (for rheumatologic and other reason)							
No	42	0.955	37	1	79	0.975	*0.189*
Yes	2	0.045	0	0	2	0.025	
Airway inflammation							
FeNO (Visit 3), ppb. Mean [SD]	37.27	24.23	24.43	14.49	31.41	21.25	*0.004*
FeNO (Visit 3), ppb. Median [IQR]	27	19–52	21	15–29.5	23	16–40.5	*0.013*
FeNO (Visit 3) ≥ 20 ppb							
No	12	27.3%	17	45.9%	29	35.8%	*0.081*
Yes	32	72.7%	20	54.1%	52	64.2%	

**Table 2:** Description of oxidative stress results and PM-OP metrics as a function of their asthma or control status.

	Asthma		Non-Asthma		All		*p* *Value*
	N = 44		N = 37		N = 81		
Oxidative stress markers							
Total ROS/RNS. μM H_2_O_2_ equival. Mean [SD]	4.94	1.33	5.89	1.06	5.37	1.30	*0.001*
Total ROS/RNS. μM H_2_O_2_ equival. Median [IQR]	5.27	2.05	5.72	1.09	5.54	1.47	*0.003*
PCC. nmol/mg. Mean [SD]	0.44	0.17	0.44	0.22	0.44	0.20	*0.869*
PCC. nmol/mg. Median [IQR]	0.41	0.27	0.47	0.24	0.42	0.25	*0.894*
HNE-OxLDL ng/mL. Mean [SD]	114,406.2	36,125.8	71,473.7	15,943.0	94,795.0	35,762.2	*<0.001*
HNE-OxLDL ng/mL. Median [IQR]	105,347.5	50,430.8	72,463.8	24,674.7	88,500.7	47,731.0	*<0.001*
8-OHdG ng/mL. Mean [SD]	13.71	11.96	10.45	5.51	12.22	9.65	*0.112*
8-OHdG ng/mL. Median [IQR]	9.98	12.90	8.81	6.07	9.69	9.16	*0.55*
GSH. Mean [SD]	3.60	2.32	3.83	1.76	3.70	2.08	*0.62*
GSH. Median [IQR]	2.86	1.30	3.40	1.13	3.21	1.17	*0.015*
PM-OP metrics (nmol/min/m^3^)							
OP-DTT PM2.5. Mean [SD]	0.30	0.29	0.17	0.25	0.24	0.27	*0.029*
OP-DTT PM2.5. Median [IQR]	0.24	0.15–0.34	0.10	0.03–0.18	0.16	0.1–0.31	*<0.001*
OP-AA PM2.5. Mean [SD]	0.72	1.29	0.34	0.89	0.55	1.14	*0.127*
OP-AA PM2.5. Median [IQR]	0.23	0.12–0.49	0.15	0.06–0.28	0.18	0.07–0.37	*0.027*
OP-DTT PM10-2.5. Mean [SD]	0.18	0.11	0.14	0.11	0.16	0.11	*0.058*
OP-DTT PM10-2.5. Median [IQR]	0.17	0.10–0.26	0.11	0.06–0.19	0.13	0.08–0.22	*0.052*
OP-AA PM10-2.5. Mean [SD]	0.59	1.38	0.17	0.11	0.40	1.04	*0.051*
OP-AA PM10-2.5. Median [IQR]	0.22	0.10–0.55	0.20	0.1–0.20	0.20	0.1–0.39	*0.029*

SD = standard deviation. IQR = interquartile rank.

**Table 3:** Crude and adjusted mean differences (MDs) between asthmatic and non-asthmatic volunteers for each of the oxidative stress markers.

	Total ROS/RNS				PCC				HNE-OxLDL				8-OHdG				GSH			
	μM H_2_O_2_ Equiv				nmol/mg				ng/mL				ng/mL				ng/mL			
Asthma–Non-Asthma	MD	95%	CI	*p* *Value*	MD	95%	CI	*p* *Value*	MD	95%	CI	*p* *Value*	MD	95%	CI	*p Value*	MD	95%	CI	*p* *Value*
Crude	−0.95	−1.49	−0.41	*0.001*	0.01	−0.08	0.10	*0.869*	42,932.44	30,170.83	55,694.06	*<0.001*	3.25	−1.00	7.50	*0.132*	−0.23	−1.16	0.69	*0.62*
Adjusted model 1	−1.13	−1.77	−0.50	*0.001*	−0.01	−0.12	0.09	*0.799*	44,353.69	29,605.35	59,102.02	*<0.001*	4.35	−0.71	9.41	*0.091*	−0.09	−1.21	1.03	*0.873*
Adjusted model 2	−1.13	−1.82	−0.44	*0.002*	0.01	−0.11	0.13	*0.887*	47,529.72	31,494.71	63,564.74	*<0.001*	5.66	0.17	11.15	*0.044*	−0.17	−1.37	1.04	*0.782*
Adjusted model 3	−1.22	−1.90	−0.55	*0.001*	0.01	−0.10	0.12	*0.876*	44,301.67	28,430.36	60,172.98	*<0.001*	4.87	−0.55	10.28	*0.078*	−0.27	−1.44	0.90	*0.645*
Adjusted model 1 + LDL *									47,234.26	12,709.81	90,870.13	*0.01*								
Adjusted model 2 + LDL *									50,059.80	35,255.70	64,863.91	<0.001								
Adjusted model 3 + LDL *									47,299.64	32,589.80	62,009.49	<0.001								

MD = mean difference asthma–non-asthma. Crude = crude MD. Adjusted model 1 = MD adjusted for age, sex, study level, and BMI according to WHO classification. Adjusted model 2 = aMD adding to model 1: FeNO levels and OP-DTT for the fine (PM2.5) and coarse (PM10-2.5) fractions. Adjusted model 3 = aMD adding to model 1: FeNO levels and OP-AA for the fine (PM2.5) and coarse (PM10-2.5) fractions. * = aMD adding total low-density lipoprotein (LDL) levels to models 1–3.

**Table 4:** Adjusted mean differences between higher PM-OP and oxidative stress levels for the total sample.

	Total ROS/RNS				PCC				HNE-OxLDL				8-OHdG				GSH			
	μM H_2_O_2_ Equiv				nmol/mg				ng/mL				ng/mL				ng/mL			
PM-OP nmol min^−1^ m^−3^	aMD	95%	CI	*p Value*	AMD	95%	CI	*p Value*	aMD	95%	CI	*p Value*	aMD	95%	CI	*p Value*	aMD	95%	CI	*p Value*
OP-DTT PM2.5	−0.32	−0.93	0.30	*0.308*	−0.03	−0.13	0.07	*0.55*	−9971.15	−23,992.72	4050.41	*0.161*	−2.14	−6.99	2.71	*0.382*	−0.26	−1.34	0.81	*0.627*
OP-AA PM2.5	−0.32	−0.89	0.24	*0.258*	−0.01	−0.11	0.08	*0.815*	−3174.65	−16,301.97	9952.66	*0.631*	1.22	−3.28	5.73	*0.59*	−0.74	−1.72	0.25	*0.141*
OP-DTT PM10-2.5	−0.11	−0.71	0.49	*0.718*	0.00	−0.10	0.10	*0.993*	−2567.63	−16,384.42	11,249.16	*0.712*	−2.46	−7.17	2.25	*0.302*	0.72	−0.32	1.76	*0.172*
OP-AA PM10-2.5	−0.02	−0.65	0.60	*0.941*	−0.04	−0.15	0.06	*0.396*	243.27	−14,100.43	14,586.98	*0.973*	−0.35	−5.27	4.57	*0.889*	0.48	−0.60	1.57	*0.379*

aMD = mean difference adjusted for asthma or control status, age, sex, educational level, and BMI according to WHO classification.

**Table 5:** Adjusted odds ratios (ORs) between higher PM-OP and oxidative stress levels for the total sample.

	Total ROS/RNS				PCC				HNE-OxLDL				8-OHdG				GSH			
	μM H_2_O_2_ Equiv				nmol/mg				ng/mL				ng/mL				ng/mL			
PM-OP nmol min^−1^ m^−3^	aOR	95%	CI	*p* *Value*	aOR	95%	CI	*p* *Value*	aOR	95%	CI	*p* *Value*	aOR	95%	CI	*p* *Value*	aOR	95%	CI	*p* *Value*
OP-DTT PM2.5	0.55	0.18	1.66	*0.291*	0.45	0.15	1.32	*0.146*	0.69	0.17	2.81	*0.606*	0.38	0.12	1.2	*0.099*	0.78	0.27	2.29	*0.654*
OP-AA PM2.5	1.17	0.42	3.25	*0.765*	0.45	0.16	1.22	*0.115*	0.85	0.24	3.05	*0.803*	0.93	0.34	2.55	*0.886*	0.87	0.33	2.35	*0.788*
OP-DTT PM10-2.5	1.59	0.54	4.71	*0.399*	1.08	0.39	2.98	*0.886*	0.93	0.25	3.46	*0.916*	0.68	0.24	1.96	*0.478*	1.66	0.58	4.77	*0.344*
OP-AA PM10-2.5	1.22	0.4	3.7	*0.727*	0.5	0.17	1.46	*0.205*	0.5	0.12	2.17	*0.355*	0.73	0.25	2.14	*0.568*	0.76	0.26	2.18	*0.607*

aOR = odds ratios adjusted for asthma or control status, age, sex, educational level, and BMI according to WHO classification.

## Data Availability

Data cannot be made publicly available in order to protect patient privacy. The data are available on request from the University of Cantabria Archive (http://repositorio.unican.es/ (accessed on 14 March 2025)) for researchers who meet the criteria for access to confidential data. Requests may be sent to the Ethics Committee (ceicc@idival.org), or Miguel Santibañez (santibanezm@unican.es).

## Supplementary Materials

The following supporting information can be downloaded at https://www.mdpi.com/article/10.3390/antiox14040385/s1. Text S1. Detailed procedures of the ELISA tests used to determine the oxidative stress biomarkers. Figure S1. Location of volunteers’ residences and the two stationary sampling points (urban and urban–industrial) used in a previous study (Expósito et al. [32]). Levels of OP-DTT and OP-AA (nmol min^−1^ m^−3^) of PM10-2.5 and PM2.5 samples are also shown on the map. Table S1. Inclusion and exclusion criteria for asthmatic patients and controls without asthma. TableS2: Visit protocol for the volunteers (n = 81). Table S3: PM-OP detection limits (D.L), mean of blank filters, and percentage of samples higher than the D.L. Table S4: Description of the oxidative stress markers used in our study, with reference to studies that have used them in blood samples. Table S5: Description of asthma patients as a function of gender.

## Nomenclature

8-OHdG	8-hydroxydeoxyguanosine
AA	ascorbic acid
ACT	asthma control test
aMDs	adjusted mean differences
aORs	adjusted odds ratios
BMI	body mass index
CIs	confidence intervals
COPD	chronic obstructive pulmonary disease
Cu	copper
DCFH	dichlorodihydrofluorescein
DTT	dithiothreitol
Fe	iron
FeNO	fractional exhaled nitric oxide
FlOPs	fluorescent oxidation products
GSH	human reduced glutathione
H_2_O_2_	hydrogen peroxide
HL	Hospital de Liencres
HNE-OxLDL	4-hydroxynonenal-modified LDL
HUMV	Hospital Universitario Marqués de Valdecilla
ICS	inhaled corticosteroids
IQR	interquartile ranges
LABAs	long-acting β adrenoceptor agonists
LDL	low-density lipoprotein
MDA	malondialdehyde
NO	nitric oxide
ONOO^-^	peroxynitrite anion
OP	oxidative potential
OS	oxidative stress
OxLDL	oxidized low-density lipoprotein
PCC	protein carbonyl content
PM	particulate matter
PM10-2.5	coarse PM fraction
PM2.5	fine PM fraction
PM-OP	oxidative potential of PM
PON-1	human serum paraoxonase-1
PTFE	polytetrafluoroethylene
RNS	reactive nitrogen species
ROO·	peroxyl radical
ROS	reactive oxygen species
SD	standard deviation
TAI	test of adherence to inhalers
