# Supplementary material for: Characterization of Systemic Oxidative Stress in Asthmatic Adults Compared to Healthy Controls and Its Association with the Oxidative Potential of Particulate Matter Collected Using Personal Samplers
Source: Antioxidants (Basel). 2025 Mar 25;14(4):385. doi: 10.3390/antiox14040385 (PMC12024361; doi:10.3390/antiox14040385)
Supplement: Supplementary file 1 [file antioxidants-14-00385-s001.zip › antioxidants-3513034-supplementary.pdf]

Supplementary material

Text S1. Detailed procedures of the ELISA tests used to determine the oxidative stress biomarkers.

**ROS/RNS μM H_2O_2 equiv**	Total ROS/RNS free radical activity was assessed using the OxiSelect™ In Vitro ROS/RNS Assay Kit (Cell Biolabs, Inc., San Diego, CA, USA) following the manufacturer's instructions. Serum samples were previously diluted in a 1:2 ratio. All reagents were prepared and combined prior to commencing the test. Initially, 50 μL of samples and hydrogen peroxide standards were introduced into the wells. Continuously, 50 μL of catalyst was added and incubated for 5 minutes at room temperature. Next, 100 μL of DCFH solution to each well. The plate was shielded from light and incubated for 15 to 45 minutes at ambient temperature. Fluorescence was ultimately measured at 480 nm excitation and 530 nm emission. A standard curve was established for each experiment; specifically, the H_2O_2 Standard Curve was employed for measuring hydrogen peroxide levels in our samples. The intra-assay coefficients for all samples varied between 0.024% and 6.26%.
PCC **nmol/mg**	PCC levels in serum samples were assessed using an OxiSelect™ Protein Carbonyl ELISA kit (Cell Biolabs, Inc., San Diego, CA, USA) following the manufacturer's instructions. A Bradford assay was employed to determine protein content of the samples. BSA standards and samples at a final concentration of 10 μg/mL were added to the wells and incubated for 2 hours at 37ºC. Subsequently, 100 μL of dinitrophenyl hydrazine (DNPH) Working Solution was added and incubated for 45 minutes at room temperature in the absence of light. After seven washes, samples were treated with 200 μL of Blocking Solution and subsequently incubated with 100 μL of diluted anti–DNP primary antibody for one hour at room temperature on an orbital shaker. Following three washes, samples were incubated with 100 μL of the diluted HRP-conjugated secondary antibody for one hour at room temperature on an orbital shaker. Ultimately, following five further washing, samples were incubated for 15 minutes with the substrate solution. To halt the enzymatic process, 100 μL of Stop Solution was added. The absorbance of the plate was measured at a wavelength of 450 nm. A standard curve was generated for each experiment. The intra-assay coefficients for all samples varied between 0.018% and 8.08%.
**HNE-OxLDL ng/mL**	Levels of 4-hydroxynonenal (HNE)-modified LDL (HNE-OxLDL was assessed using the OxiSelect™ Human Oxidized LDL ELISA Kit (HNE-LDL Quantitation) (Cell BioLabs, San Diego, CA, USA) following the manufacturer's instructions. Serum samples were initially diluted at a ratio of 1:25, followed by an additional dilution of 1:8 as per the protocol's recommendations. A volume of 100 μL of OxLDL standard and serum sample were introduced to the coated plate and incubated for two hours on an orbital shaker. Plates were washed thrice, followed by the addition of 100 μL of Blocking Reagent, which was incubated for 1 hour on an orbital shaker. Subsequent to five more washes, wells were treated with 100 μL of Biotinylated Anti-Human ApoB-100 antibody and incubated for one hour on an orbital shaker. Subsequently, five further washes were conducted, and 100 μL of Streptavidin-Enzyme Conjugate was added and incubated for one hour on an orbital shaker. Subsequently, five further washes were conducted, and 100 μL of Substrate Solution was added and incubated for a duration of 2 to 30 minutes. The

	reaction was terminated using 100 μL of Stop Solution. Absorbance was quantified at a wavelength of 450 nm. A standard curve was generated for each experiment. The intra-assay coefficients for all samples varied between 0.007% and 4.68%.
**8-OHdG** **ng/mL**	Levels of 8-OHdG were assessed using the OxiSelect™ Oxidative DNA Damage ELISA Kit (Cell BioLabs, San Diego, CA, USA) following the manufacturer's instructions. Serum samples were previously diluted at a ratio of 1:20. Initially, plates were treated with an 8-OHdG-BSA conjugate and incubated overnight at 4ºC. The following day, the plates were washed and incubated for 1 hour at room temperature with assay diluent. Subsequently, 50 μL of the sample or 8-OHdG standards were added and incubated for 10 minutes in an orbital shaker. An extra 50 μL of anti-8-OHdG monoclonal antibody was added and incubated for 1 hour in an orbital shaker. Subsequent to three washing cycles, 100 μL of secondary HRP-conjugated antibody was added and incubated for an additional hour on an orbital shaker. Subsequently, three further wash processes were conducted, followed by the addition of 100 μL of a colorimetric substrate solution, which was incubated for 2 to 30 minutes. The reaction is subsequently halted, and the absorbance is quantified at 450 nm. The intra-assay coefficients for all samples ranged from 0.004% to 3.80%.
**GSH** **ng/mL**	Levels of GSH were assessed using the Human Reduced Glutathione ELISA Kit (MyBioSource, San Diego, CA, USA) following the manufacturer's instructions. All kit components and serum samples were equilibrated to room temperature prior to initiating the experiment. Initially, 100 μL of the standards and samples were dispensed into the plate and treated with 50 μL of horseradish (HRP)−conjugate reagent for one hour at 37ºC. Subsequently, the plate underwent five washes with Wash Solution. Afterwards, 50 μL of chromogen solutions A and B were included. Plates were incubated for 15 minutes at 37ºC protected from light. The reaction was terminated with 50 μL of Stop Solution and the absorbance was measured at a wavelength of 450 nm. A standard curve was plotted with every assay. The intra-assay coefficients for all samples varied between 0.09% and 6.49%.

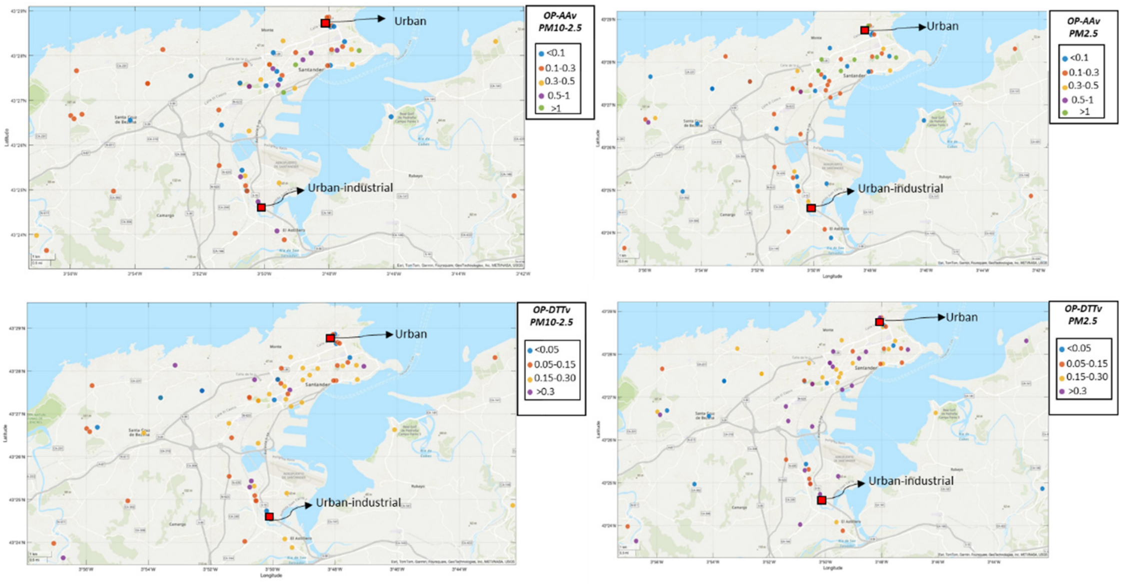

Figure S1. Location of volunteers' residences and the two stationary sampling points (urban and urban-industrial) used in a previous study (Expósito et al. [32]). Levels of OP-DTT and OP-AA (nmol min^{-1} m^{-3}) of PM10-2.5 and PM2.5 samples are also shown in the map.

Table S1. Inclusion and exclusion criteria for asthmatic patients and controls without asthma.

**Asthmatic patients**
Inclusion criteria:<br>1) Diagnosis of asthma according to GINA criteria (GINA Guideline, 2020), at least 12 months prior to the baseline visit.<br>2) Stable treatment with inhaled corticosteroids (ICS) with/without long-acting β adrenoceptor agonists (LABAs), the previous 3 months.<br>3) No exacerbations in the 4 weeks prior to study inclusion.<br>4) No smoker or former smoker >12 months<br>Exclusion criteria<br>1) Previous diagnosis of confirmed chronic obstructive pulmonary disease (COPD)<br>2) Being treated with oral steroids for other reasons than asthma were exclusion criteria.
**Controls without asthma**
Inclusion criteria<br>1) No smoker or former smoker >12 months<br>2) Without chronic or recurrent respiratory symptoms or features typical of asthma<br>Exclusion criteria<br>1) Previous diagnosis of confirmed asthma<br>2) Being treated with oral or inhaled corticosteroids or with biologic therapy for any reason.

Table S2. Visit protocol

**Day 1. Visit 1.**	**Day 2 (lag0). Visit 2.**	**Day3 (lag1). Visit 3. day 3** (25-48 hours after returning the personal monitor)
Informed consent form<br>Questionnaire<br>PM personal monitor delivery	Return of the PM personal monitor<br>Questionnaire (summary of activities done with the PM personal monitor)	-Blood collection 8:00-9:00h for 1) blood count, conventional lipid profile and glucose, 2) oxidative stress markers, 3) IL6 and IL10 determinations<br>-FeNO

Table S3. PM-OP detection limits (D.L), mean of blank filters, and percentage of samples higher than the D.L.

	**Blank mean (µM/min)**		**D.L. (µM/min)**		**% of n>D.L.**	
	**Asthma**	**Control**	**Asthma**	**Control**	**Asthma**	**Control**
OP-DTT PM2.5	0.10	0.11	0.049	0.042	92	68
OP-AA PM2.5	0.23	0.12	0.081	0.034	73	78
OP-DTT PM 10-2.5	0.10	0.12	0.036	0.036	86	78
OP-AA PM10-2.5	0.21	0.11	0.051	0.022	73	89

Table S4. Description of the oxidative stress markers used in our study, with reference to studies that have used them in blood samples.

**Oxidative stress marker**	**Indicator of**	**ELISA KIT** (catalog number)	**Intra-assay coefficient range**	**Lowest-highest quantifications**	**References***
In Vitro reactive oxygen species (ROS)/ reactive nitrogen species (RNS)	Total ROS/RNS content	STA-347	0.024% to 6.26%	1.69 to 8.85 µM H_2O_2 equivalents	[34,35]
Protein carbonyl content (PCC)	Protein damage	STA-310	0.018% and 8.08%	0.05 to 1.24 nmol/mg protein	[36-40]
4-hydroxynonenal - Oxidized low density lipoprotein (HNE-OxLDL)	Lipid peroxidation	STA-389	0.007% and 4.68%	46904.38 to 216985.40 ng/mL	[1,36,41,42]
8-hydroxydeoxyguanosine (8-OHdG)	DNA/RNA damage and repair	STA-320-T	0.004% to 3.80%	0.61 to 47.84 ng/mL	[36,42-44]
Reduced glutathione (GSH)	Antioxidants & antioxidant capacity	MBS727656	0.09% to 6.49%	1.76 to 14.24 ng/mL	[36-39,43,45]

*references are numbered in relation to their order in the main article.

Table S5. Description of asthmatic patients as a function of gender.

	Women Asthma N=25		Men Asthma N=19		All Asthma N=44		*p value*
FEV1%. Mean [SD]	89.04	16.883	97.21	17.018	92.57	17.24	*0.122*
FEV1%. Median [IQR]	91	77-105.5	92	87-106	91	82-105.8	
ACT. Mean [SD]	22.04	4.24	22.32	3.23	22.16	3.8	*0.815*
ACT. Median [IQR]	23	20.5-25	23	20-25	23	20.3-25	*0.98*
ACT≤19 Uncontrolled asthma	5	20.0%	3	15.8%	8	18.2%	*0.72*
ACT≥20 Controlled asthma	20	80.0%	16	84.2%	36	81.8%	
Number of moderate-severe exacerbations without hospital admission (previous 12 months)							
0	17	68.0%	17	89.5%	34	77.3%	*0.107*
1	4	16.0%	1	5.3%	5	11.4%	
2	4	16.0%	0		4	9.1%	
4	0		1	5.3%	1	2.3%	
Number of severe exacerbations with hospital admission (previous 12 months)							
0	23	92.0%	18	94.7%	41	93.2%	*0.668*
1	1	4.0%	1	5.3%	2	4.5%	
2	1	4.0%	0		1	2.3%	
Number of severe exacerbations with ICU admission (previous 12 months)							
0	25	100.0%	19	100.0%	44	100.0%	
Need for systemic corticosteroids							
No	17	68.0%	16	84.2%	33	75.0%	*0.219*
Yes	8	32.0%	3	15.8%	11	25.0%	
TAI 10 Items. Mean [SD]	49.64	0.81	49.21	1.55	49.45	1.19	*0.24*
TAI 10 Items. Median [IQR]	50	50-50	50	49-50	50	49.25-50	*0.349*
Low adherence (≤49 puntos)	5	20.0%	6	31.6%	11	25.0%	*0.38*
Poor adherence (≤45 points)	0		1	5.3%	1	2.3%	*0.428*
Intermediate adherence (46-49 points)	5	20.0%	5	26.3%	10	22.7%	
Good adherence (50 points)	20	80.0%	13	68.4%	33	75.0%	
GINA stage							
3	4	16.0%	1	5.3%	5	11.4%	*0.514*
4	13	52.0%	12	63.2%	25	56.8%	
5	8	32.0%	6	31.6%	14	31.8%	
GEMA stage							
3	4	16.0%	1	5.30%	5	11.4%	*0.641*
4	10	40.0%	8	42.1%	18	40.9%	
5	7	28.0%	5	26.3%	12	27.3%	
6	4	16.0%	5	26.3%	9	20.5%	
Biologic treatment							
No	21	84.0%	14	73.7%	35	79.5%	*0.401*
Yes	4	16.0%	5	26.3%	9	20.5%	
FeNO≥20ppb							
No	8	32.0%	4	21.1%	12	27.3%	*0.419*
Yes	17	68.0%	15	78.9%	32	72.7%	
Blood Eosinophils ≥150 cells/mm^3							
No	6	24.0%	6	31.6%	12	27.3%	*0.576*
Yes	19	76.0%	13	68.4%	32	72.7%	
Blood Neutrophils ≥5000 cells/mm^3							
No	22	88.0%	15	78.9%	37	84.1%	*0.416*
Yes	3	12.0%	4	21.1%	7	15.9%	
Prick test							
Negative	16	64.0%	11	57.9%	27	61.4%	*0.68*
Positive at least in one antigen	9	36.0%	8	42.1%	17	38.6%	
IgE ≥250 IU/ml							
No	20	80.0%	10	52.6%	30	68.2%	*0.054*
Yes	5	20.0%	9	47.4%	14	31.8%	

ACT= Asthma control test. TAI= Test of adherence to inhalers. GINA= Global initiative for Asthma. Global Strategy for Asthma Management and Prevention, 2023. GEMA= Spanish Guideline on the Management of Asthma v5.3.
